# Effect of Small Molecules on Blastocyst Development and Outgrowth Establishment of Bovine Haploid Parthenogenetic Embryos

**DOI:** 10.3390/ani16101517

**Published:** 2026-05-15

**Authors:** Luis Aguila, Rodrigo Castillo, Felipe Pérez-García, Favian Treulen, Cecilia Valencia, Felipe Perecin, Lawrence Charles Smith, Maria Elena Arias, Ricardo Felmer

**Affiliations:** 1 Laboratory of Reproduction, Centre of Reproductive Biotechnology (CEBIOR-BIOREN), Faculty of Agriculture and Environmental Sciences, Universidad de La Frontera, Temuco 4811322, Chile; rodrigo.acastillok@gmail.com (R.C.); f.perez12@ufromail.cl (F.P.-G.); valencia.robles.cecilia@gmail.com (C.V.); mariaelena.arias@ufrontera.cl (M.E.A.); 2 Escuela de Tecnología Médica, Facultad de Medicina y Ciencias de la Salud, Universidad Mayor, Temuco 4801043, Chile; favian.treulen@umayor.cl; 3 Departamento de Medicina Veterinária, Faculdade de Zootecnia e Engenharia de Alimentos, Universidade de São Paulo, Pirassununga 13635-900, Brazil; fperecin@usp.br; 4 Centre de Recherche en Reproduction et Fértilité (CRRF), Université de Montréal, Saint-Hyacinthe, QC J2S 2M2, Canada; lawrence.c.smith@umontreal.ca

**Keywords:** uniparental embryos, haploidy, bovine IVF, embryonic outgrowths, serum-free culture

## Abstract

Haploid embryos constitute a valuable model for genetic and epigenetic studies; however, their developmental competence is reduced compared with diploid counterparts. This study evaluated whether supplementation of the culture medium with specific small molecules could improve developmental competence and outgrowth establishment of parthenogenetic haploid embryos. The effects of TGF-β inhibition (A83-01), WNT pathway modulation (CHIR99021 and IWR-1), and activin A (AA) supplementation were assessed from the morula stage onward under serum-free conditions. A83-01 treatment did not improve blastocyst formation or morphology and was associated with reduced total cell numbers relative to IVF controls. CHIR99021 supplementation increased the number of SOX2-positive cells compared with IWR-1 and vehicle-treated embryos, suggesting partial support of pluripotency; however, overall developmental progression remained inferior to diploid controls. In contrast, activin A significantly increased the proportion of haploid morulae developing into blastocysts and improved hatching rates. Nevertheless, AA supplementation did not restore CDX2-positive cell numbers or total cell counts to diploid levels. Furthermore, neither CHIR99021 nor AA affect DNA fragmentation levels, although a tendency toward increased TUNEL-positive cells was observed. Activin A treatment also failed to improve embryonic outgrowth formation. Collectively, these findings demonstrate that although activin A enhances blastocyst yield and hatching in bovine haploid embryos, modulation of TGF-β or WNT signaling alone is insufficient to restore diploid-like proliferative developmental competence.

## Simple Summary

Haploid embryos constitute a valuable model for genetic and epigenetic studies; however, their developmental competence is reduced compared with diploid counterparts. This study evaluated whether supplementation of the culture medium with specific small molecules could improve developmental competence and outgrowth establishment of parthenogenetic haploid embryos. The effects of TGF-β inhibition (A83-01), WNT pathway modulation (CHIR99021 and IWR-1), and activin A (AA) supplementation were assessed from the morula stage onward under serum-free conditions. A83-01 treatment did not improve blastocyst formation or morphology and was associated with reduced total cell numbers. CHIR99021 supplementation increased the number of SOX2-positive cells compared with IWR-1 and vehicle-treated embryos, suggesting partial support of pluripotency; however, overall developmental progression remained inferior to diploid controls. In contrast, activin A significantly increased the proportion of haploid morulae developing into blastocysts and improved hatching rates. Nevertheless, AA supplementation did not restore cell counts to diploid levels. Furthermore, neither CHIR99021 nor AA affect DNA fragmentation levels, although a tendency toward increased levels was observed. Activin A treatment also failed to improve embryonic outgrowth formation. Collectively, these findings demonstrate that although activin A enhances blastocyst yield and hatching in bovine haploid embryos, modulation of TGF-β or WNT signaling does not substantially influence the developmental competence or molecular features of haploid parthenogenetic embryos comparable to those of diploid or biparental embryos.

## 1. Introduction

Biparental embryonic development in mammals requires contributions from both the maternally and paternally inherited haploid genomes. However, early development can be achieved from uniparental embryos in mammals using artificial oocyte activation and/or micromanipulation techniques, and these have been extremely useful in delineating genomic function, imprinting status and parental-specific roles in ontogenesis [1,2].

Uniparental embryos contain exclusively maternal or paternal genomes. These embryos can be classified according to their genomic origin as well as their ploidy. Parthenogenetic embryos contain only oocyte-derived (maternal) chromosomes and their counterpart androgenetic embryos contain only sperm-derived (paternal) chromosomes. According to their ploidy, they can be diploid (two sets of chromosomes), haploid (one set of chromosomes) or polyploid (>2 sets of chromosomes).

Haploid embryos are efficient models for genome imprinting research and allow studies on the contribution of the paternal and maternal genomes to early embryonic development [3]. Moreover, haploid embryos have been used to derive embryonic stem cells and hold great promise for functional genetic studies and animal biotechnology [4,5].

Although haploid embryonic stem cells (hESCs) have been obtained in several mammals [6,7,8], most reports have indicated poor rates of blastocyst formation, indicating constraints at early stages of embryonic development. In mice, studies have revealed that the preimplantation developmental potential of haploids is significantly impaired relative to diploid embryos, due mainly to the disruption of gene regulatory mechanisms [9,10] and abnormal imprinted gene expression [11,12,13]. However, studies investigating these limitations in bovine uniparental embryos remain scarce.

One possible strategy to enhance in vitro competence relies on modifications of the culture medium, for example, by supplementing it with small molecules capable of modulating pathways associated with early developmental potential. In this context, the use of small molecules to activate or inhibit the WNT and MEK/ERK signaling pathways influences critical processes associated with apoptosis and implantation [14,15]. For instance, the study of [16] found that Activin A supplementation stimulates core pluripotent factors, such as NANOG, during self-renewal of bovine embryonic stem cells (ESC), while reducing SRY-box transcription factor 2 (SOX2), a key epiblast-associated factor, at the blastocyst stage, and increasing the proportion of cells expressing caudal-type homeobox 2 (CDX2), a trophectoderm-associated marker. Additionally, supplementation of the culture medium with PD0325901 and CHIR99021 (MEK inhibition and WNT activation, respectively) has been shown to accelerate blastocyst development and increase both inner cell mass (ICM) and trophoblast cell numbers, alongside elevated expression of epiblast markers such as NANOG and SOX2 [17]. Similarly, combined inhibition of TGF-β signaling, GSK3β, and MEK1/2 demonstrated to support robust in vitro self-renewal and maintenance of pluripotency in bovine induced pluripotent stem cells [18]. Therefore, targeted regulation of pluripotency states emerges as a promising strategy to enhance the developmental potential of embryos in vitro, particularly in large animal species.

Therefore, the objective of the present study was to evaluate the effect of the supplementation with small molecules in the embryo culture medium on developmental competence and the establishment of outgrowths from morula stage bovine parthenogenetic haploid embryos. The findings indicate that while pathway modulation may influence lineage allocation dynamics, it is insufficient to overcome the broader developmental constraints associated with uniparental haploidy in bovine species.

## 2. Materials and Methods

### 2.1. Oocyte Collection and In Vitro Maturation

Bovine ovaries were obtained from cows and heifers at a local slaughterhouse and transported to the laboratory in sterile 0.9% NaCl maintained at 25–30 °C in a thermos bottle. Cumulus–oocyte complexes (COCs) were aspirated from 2–8 mm antral follicles using an 18-gauge disposable needle and pooled in a 50 mL conical tube. For in vitro maturation (IVM), COCs with multiple layers of cumulus cells were selected, washed, and transferred in groups of 50 complexes to a 4-well culture plate containing 500 μL of maturation medium per well. Maturation medium consisted of TCM199 (Invitrogen Life Technologies, Waltham, MA, USA), supplemented with 10% fetal bovine serum (Invitrogen Life Technologies), 0.2 mM pyruvate (Sigma-Aldrich, St. Louis, MO, USA), 25 mg/mL gentamicin (Sigma-Aldrich), 6 μg/mL luteinizing hormone (Sioux Biochemica, Inc., Sioux Center, IA, USA), 6 μg/mL follicle-stimulating hormone (Bioniche Life Science, Inc., Belleville, ON, Canada), and 1 μg/mL estradiol (Sigma-Aldrich). In vitro oocyte maturation was carried out for 22–24 h at 38.5 °C in a humidified atmosphere at 5% CO_2_.

### 2.2. In Vitro Fertilization

In vitro fertilization (IVF): After 22–24 h of IVM, COCs were washed twice in IVF medium before being transferred in groups of 50 to a 4-well culture plate containing 350 μL of medium per well. The IVF medium consisted of modified Tyrode’s lactate medium was used containing the following components: 126 mM NaCl, 4.7 mM KCl, 0.4 mM MgSO_4_, 0.3 mM NaH_2_PO_4_, 22.4 mM sodium lactate, 1 mM pyruvic acid sodium salt, 2 mM CaCl_2_, and 15 mM NaHCO_3_, and supplemented with fatty-acid-free BSA (0.6% *w*/*v*), pyruvate (0.2 mM), heparin (2 μg/mL), and gentamicin (50 mg/mL). COCs were transferred to a 4-well culture plate 15 min prior to adding the spermatozoa. To stimulate sperm motility, penicillamine (2 mM; Sigma-Aldrich), hypotaurine (1 mM; Sigma-Aldrich), and epinephrine (250 mM; Sigma-Aldrich) were added to each well. Motile spermatozoa were diluted with IVF medium to a final concentration of 1 × 10^6^ sperm/mL, and 16 μL of the suspension was added to each well containing the matured COCs. Fertilization was conducted at 38.5 °C for 18 h under 5% CO_2_ in a humidified atmosphere. Presumptive zygotes were denuded by vortexing.

### 2.3. Production of Parthenogenetic Embryos

Bovine haploid (hPE) and diploid (dPE) embryos were produced according to Valencia et al. [19]. Briefly, chemical oocyte activation was performed between 22 and 24 h after IVM by 5 min exposure to 5 μM ionomicyn (Calbiochem, San Diego, CA, USA). To generate haploid parthenotes, oocytes were first treated with ionomycin and then incubated for 5 h in potassium simplex optimization medium (KSOM; EmbryoMax^®^, Millipore Corp., Billerica, MA, USA) supplemented with 10 mg/mL of cycloheximide (CHX; Sigma-Aldrich). For diploid parthenote production, 6-dimethylaminopurine (DMAP) was added at a concentration of 1.9 mM in place of CHX. Following parthenogenetic activation, the presumptive zygotes were washed in HECM-HEPES (HH) medium and transferred into in vitro culture droplets.

### 2.4. In Vitro Culture

Groups of 20–25 zygotes were cultured in 40 µL droplets of EmbryoMax^®^ KSOM (Merck, Darmstadt, Germany) supplemented with 1% (*v*/*v*) Basal Medium Eagle (BME) essential amino acids (Gibco, Douglas County, IL, USA) and 1% (*v*/*v*) Minimum Essential Medium (MEM) non-essential amino acids (Gibco) and covered with embryo-tested mineral oil. In some experiments, the culture medium was supplemented either with fetal bovine serum (FBS) or insulin–transferrin–selenium (ITS; Sigma-Aldrich). Cultures were maintained at 38.5 °C under a gas atmosphere of 5% CO_2_, 5% O_2_, and 90% N_2_ at 100% humidity.

At day 5, morula-stage embryos were selected and transferred to culture medium supplemented with different small molecules: A83-01 (10 µM; Tocris Bioscience, Bristol, UK), CHIR99021 (3 µM; Sigma-Aldrich), IWR1 (2.5 µM; Sigma-Aldrich), or activin A (AA; 20 ng/mL; R&D Systems, Minneapolis, MN, USA) for 72 h, until day 8 post-activation/insemination. The concentrations used were selected based on previous studies reporting effective modulation of the corresponding signaling pathways during early embryonic development [20,21,22,23,24].

Embryonic development was assessed at the cleavage stage on day 2 (48 h), at the morula stage on day 5 (120 h) and at the blastocyst stage on day 8 (192 h) post-activation/insemination.

### 2.5. Derivation and Culture of Bovine Embryonic Outgrowths

Bovine blastocysts at day 8 were used to derive embryonic outgrowths. Unhatched blastocysts were treated with 2 mg/mL pronase (Merck Millipore, Burlington, MA, USA; 10165921001) at 38.5 °C for 5 min to remove the zona pellucida (ZP), followed by three washes in TCM-Hepes medium. ZP-free blastocysts were transferred to a 4-well plate previously precoated with 0.5 µg/cm^2^ Vitronectin (Gibco, Douglas County, IL, USA), which was then replaced with mTeSR-plus media (STEMCELL Technologies Inc., Vancouver, BC, Canada; 100-0276) supplemented with 2.5 μM IWR1 (AOBIOUS Inc., Gloucester, MA, USA; AOB33702), 2.0 μM iDOT1L (AOBIOUS Inc., Gloucester, MA, USA; AOB1922), 1.0 μM PD184352 (ApexBio Technology, Houston, TX, USA; A1792), 2.0 μM SU5402 (ApexBio Technology, Houston, TX, USA; A3843), 1.5 μM CHIR99021 (AOBIOUS Inc., Gloucester, MA, USA; AOB3866), 5 μM Forskolin (AOBIOUS Inc., Gloucester, MA, USA; AOB6380), and 20 ng/mL human LIF (R&D Systems, Minneapolis, MN, USA; 7734LF025), incubated at 37 °C and 5% CO_2_ for 24 h without disruption [25].

### 2.6. Immunostaining

Immunostaining was performed as described previously [26]. Three to eight day-8 blastocyst-stage embryos (biological replicates) per group were collected, washed in PBS with PVA, fixed with 4% paraformaldehyde for 15 min, and permeabilized with D-PBS with 1% Triton X-100 for 30 min. After blocking for 2 h in D-PBS with 0.1% Triton X-100, 1% BSA, and 5% goat serum (Gibco, Grand Island, NY, USA), the embryos were placed in a primary antibody solution composed of blocking buffer. Anti-Sox2 (Cell Signaling Technology, Danvers, MA, USA; L1D6A2) and anti-NANOG (Cell Signaling 1E6C4) mouse antibodies and anti-Cdx2 (Abcam, Cambridge, UK; ab227201) and anti-GATA3 (Cell Signaling D13C9) rabbit antibodies were used at a 1:300 dilution overnight at 4 °C. After washing 3× for 10 min and 3× for 20 min each, embryos were incubated with secondary antibodies (1:300): Alexa Fluor 633-conjugated goat anti-rabbit IgG (Invitrogen Life Technologies, Waltham, MA, USA; A-21070) and Alexa Fluor 488-conjugate goat anti-mouse IgG (Invitrogen A-11001) both at RT for 1 h. Finally, embryos were washed 3× for 10 min and mounted on slides with Prolong Gold Antifade with DAPI (Invitrogen Life Technologies, Waltham, MA, USA, cat. # P36935) and evaluated using confocal microscopy.

### 2.7. TUNEL Assay

Embryos were incubated with labeling reagents according to the manufacturer’s instructions (In Situ Cell Death Detection Kit, Fluorescein, Roche Applied Science, Indianapolis, IN, USA). A positive control for TUNEL was carried out by treating embryos with 75.4 U DNase I for 15 min at 37 °C before the TUNEL assay, and a negative control was attained by incubating embryos with the fluorescent labeling reagent in the absence of the terminal transferase dUTP enzyme. Then, to stain the cytoskeleton, embryos were incubated for 30 min with 1× phalloidin 633 (Invitrogen), according to the manufacturer’s instructions. Finally, embryos were mounted onto a glass slide with Prolong Gold Antifade with DAPI (Life Technologies, Eugene, OR, USA, cat. number P36935) and evaluated using confocal microscopy.

### 2.8. Image Acquisition and Analysis

Image acquisition and analysis were performed as previously described. Briefly, five confocal optical sections were acquired from the nucleus of each cell and processed using maximum-intensity projection of the Z-stack. Imaging was conducted with an Olympus FV1000 laser-scanning confocal microscope (Olympus Corporation, Tokyo, Japan) at the Scientific and Technological Bioresource Nucleus, Universidad de La Frontera (UFRO), Temuco, Chile. Image analysis was carried out using ImageJ software (v1.48; National Institutes of Health, NIH, Bethesda, MD, USA), available in https://imagej.nih.gov/ij/download.html, accessed on 11 May 2026.

Cell quantification was performed using ImageJ software (NIH, USA). Digital images were first converted into an RGB stack to separate the color channels, and the channel providing the highest contrast between the cells and background was selected for analysis. A threshold was then applied to segment the cells from the background. Particle detection was carried out using the “Analyze Particles” function, with size and circularity parameters adjusted to exclude background noise and debris. Detected objects were visualized using the “Outlines” option, and the total number of cells per image was automatically recorded. All images were analyzed under identical threshold and particle analysis settings to ensure consistency across samples.

### 2.9. RNA Extraction and RT-PCR

For analysis of gene expression, days 6 and 7 morula-stage embryos were pooled in groups of five embryos. Blastocysts were analyzed individually. Analysis of each group was carried out in at least three biological replicates, and each replicate was run in duplicate. Total RNA was extracted using the Arcturus PicoPure RNA Isolation kit (Life Technologies) and reverse transcribed into cDNA using SuperScript Vilo (Invitrogen). Semi-quantitative RT-PCR was performed using the RotorGene SyBr Green PCR kit (Qiagen, Hilden, Germany) in a Rotorgene Q PCR cycler under the following amplification conditions: 95 °C for 5 min, followed by 40 cycles at 95 °C for 5 s and at 60 °C for 10 s. Primers were designed using Oligo6 software and the geometric means of three reference genes (*GAPDH*, *ACTB*, and *SF3A*) were used for normalization. The stability of the reference genes across our samples was confirmed using Bestkeeper [27]. A list of all primers used can be found in Supplementary Materials (Table S1).

### 2.10. Statistical Analysis

Quantitative data sets are presented as means and standard deviation (±SD) and were analyzed using Student’s *t*-test, followed by Fisher’s least significant difference (LSD) post hoc test or one-way ANOVA, followed by Tukey post hoc test when appropriate. Binomial data sets, such as pronuclear formation, were analyzed by using Fisher’s exact test. Differences were considered significant at *p* < 0.05.

## 3. Results

### 3.1. Serum-Free Culture System

**Figure 1:**
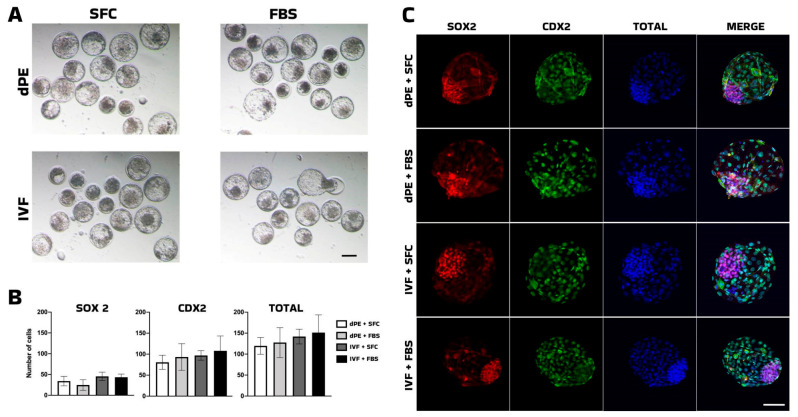
Morphological and pluripotency characteristics in blastocyst. (**A**): Representative image of bovine in vitro-fertilized (IVF) and diploid parthenogenetic (dPE) blastocyst-stage embryos cultured in serum-free culture (SFC) or in the presence of bovine fetal serum (FBS). (**B**): Barplot of quantification of pluripotency markers analyzed by immunofluorescence in blastocyst according to cell allocation and total cell number. (**C**): Representative immunofluorescent images of pluripotency markers (SOX2 and CDX2) in bovine embryos cultured in serum-free culture (SFC) or in presence of bovine fetal serum (FBS). dPE: diploid parthenogenetic embryos; IVF: in vitro-fertilized embryos. SOX2: red fluorescence channel depicting SRY-box transcription factor 2 (SOX2)-positive cells; CDX2: green fluorescence channel depicting Caudal-type homeobox 2 (CDX2)-positive cells; TOTAL: blue fluorescence channel indicating the total number of cells; Merge: composite images showing total cell number (blue), SOOX2-positive cells (red), and CDX2-positive cells (green). Scale bar = 100 μm.

To establish a serum-free culture system, we evaluated the effects of replacing FBS with insulin–transferrin–selenium (ITS) supplementation on the developmental competence of IVF-derived and diploid parthenogenetic embryos. No significant differences were observed between groups (*p* > 0.05) in cleavage rate, blastocyst formation, blastocyst morphology, total cell number, or the expression of pluripotency-associated transcription factors (Table 1, Figure 1).

Based on these findings, the ITS-supplemented medium was selected as the basal culture system for subsequent experiments aimed at assessing the effects of small-molecule modulators on embryonic competence.

### 3.2. Effects of Small Molecule Supplementation

**Figure 2:**
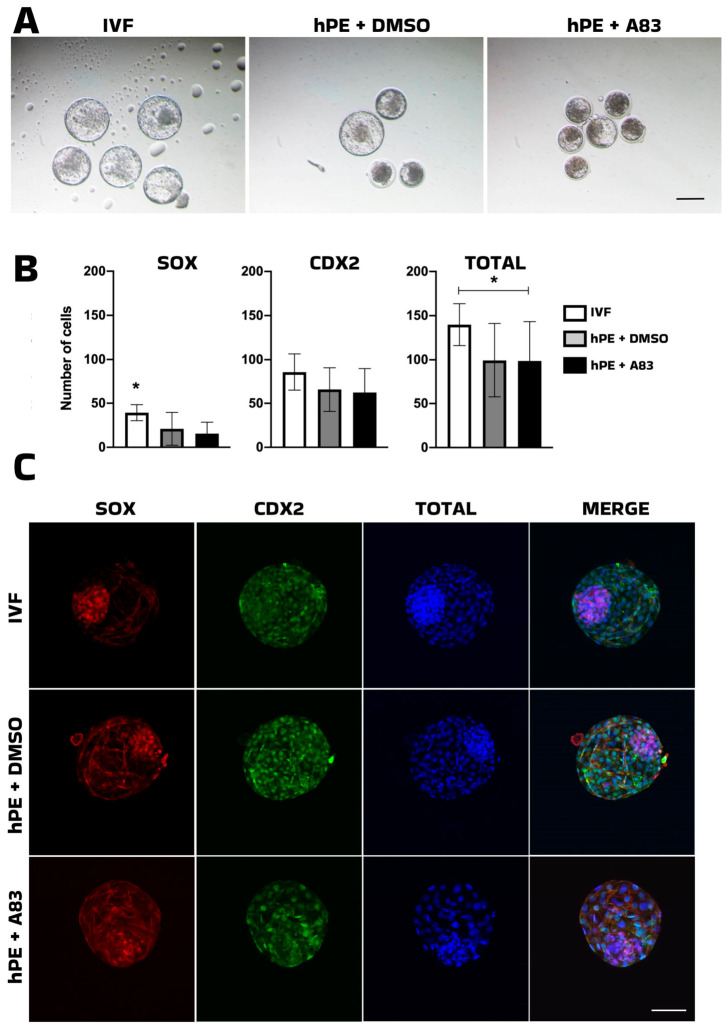
Effect of TGF-ß pathway inhibition on pluripotency markers. (**A**) Representative images of bovine in vitro-fertilized cells (IVF) and haploid parthenogenetic blastocysts (hPE) cultured in the presence of the A-83-01 (A83) inhibitor or vehicle control (DMSO) from the morula stage onward. (**B**) Bar plot showing the quantification of pluripotency marker analyzed by immunofluorescence in blastocysts, expressed according to cell lineage allocation and total cell number (**C**) Representative fluorescent images of bovine IVF and hPE embryos at the blastocyst stage. IVF: in vitro-fertilized embryos; hPE: haploid parthenogenetic embryos; DMSO: dimethyl sulfoxide; A83: A-83-01 (ALK inhibitor). SOX2: red fluorescence channel depicting SRY-box transcription factor 2 (SOX2)-positive cells; CDX2: green fluorescence channel depicting Caudal-type homeobox 2 (CDX2)-positive cells; TOTAL: blue fluorescence channel indicating the total number of cells; Merge: composite images showing total cell number (blue), SOOX2-positive cells (red), and CDX2-positive cells (green). * Indicates statistically significant differences (* *p* < 0.05). Scale bar = 100 μm.

Given previous reports indicating that inhibition of TGF-β signaling enhances pluripotency [21], we next investigated the effects of A83-01, a selective SMAD-dependent TGF-β pathway inhibitor, when applied on day 5 from the morula stage onward on the developmental competence of haploid parthenogenetic embryos. In line with earlier observations, A83-01 treatment did not alter the proportion of morulae developing to the blastocyst stage or blastocyst morphology compared with the vehicle control (DMSO) (Table 2, Figure 2).

However, when pluripotency markers and cell numbers were analyzed relative to an IVF control group, haploid parthenogenetic embryos displayed a reduced number of SOX2-positive cells under both treatment conditions (*p* < 0.05), as well as a lower total cell number in A83-01-treated embryos (*p* = 0.04) compared with IVF-derived blastocysts (Table 3, Figure 2).

Taken together, these results indicate that exposure to A83-01 from the morula stage to the blastocyst stage does not improve developmental competence or cell allocation in bovine hPE.

**Figure 3:**
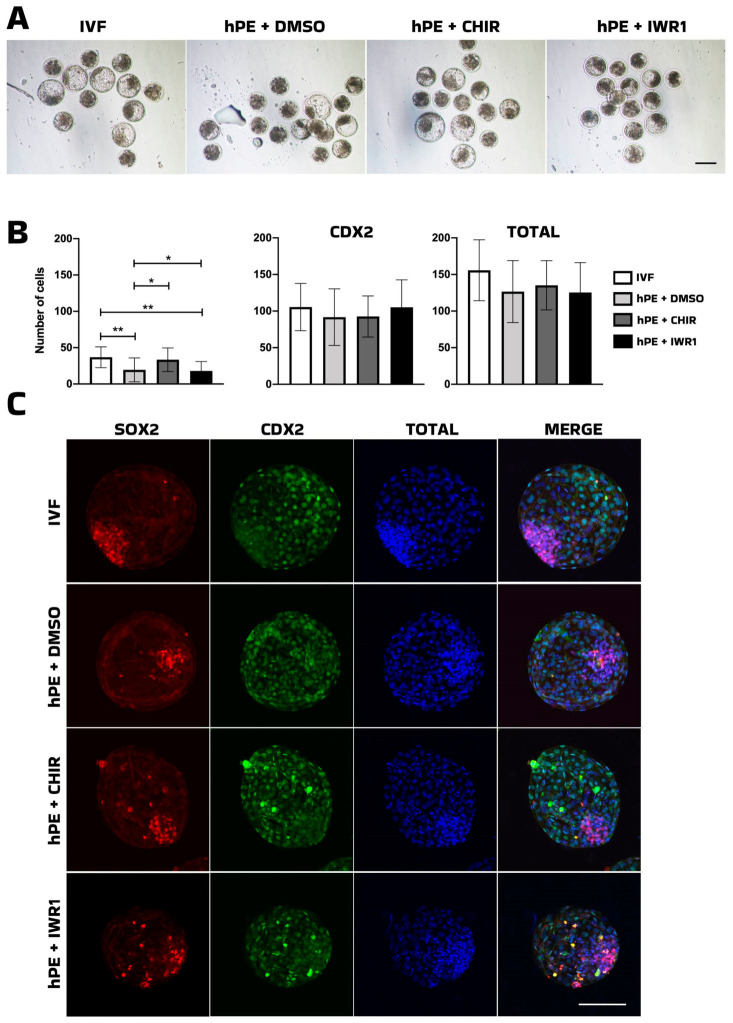
Effects of WNT pathway modulators on pluripotency markers. (**A**) Representative images of bovine in vitro-fertilized (IVF) and haploid parthenogenetic blastocysts (hPE) cultured in the presence of CHIR99021, IWR-1, or vehicle control (DMSO) from the morula stage onward. (**B**) Bar plot showing the quantification of pluripotency marker-positive cells analyzed by immunofluorescence in blastocysts, expressed according to cell lineage allocation and total cell number. (**C**) Representative fluorescent images of bovine IVF and hPE embryos at the blastocyst stage. IVF: in vitro-fertilized embryos; hPE: haploid parthenogenetic embryos; DMSO: dimethyl sulfoxide; CHIR: CHIR99021 (GSK-3 inhibitor); IWR-1: WNT signaling inhibitor. SOX2: red fluorescence channel depicting SRY-box transcription factor 2 (SOX2)-positive cells; CDX2: green fluorescence channel depicting Caudal-type homeobox 2 (CDX2)-positive cells; TOTAL: blue fluorescence channel indicating the total number of cells; Merge: composite images showing total cell number (blue), SOOX2-positive cells (red), and CDX2-positive cells (green). * Indicates statistically significant differences (* *p* < 0.05; ** *p* < 0.01). Scale bar = 100 μm.

Next, we evaluated supplementation with two modulators of the WNT signaling pathway: CHIR99021, a glycogen synthase kinase-3 beta (GSK3β) inhibitor that functions as a pathway agonist, and IWR-1, a Tankyrase-1/2 inhibitor that acts as a pathway antagonist. Cleavage rates were comparable between IVF-derived and parthenogenetic embryos (approximately 80%). However, development to the morula and blastocyst stages was significantly higher (*p* < 0.05) in IVF embryos (33% and 27%, respectively) than in parthenogenetically activated embryos (23% and 15%, respectively) (Table 4, Figure 3A).

In addition, the proportion of morulae progressing to the blastocyst stage was higher in the IVF group than in haploid embryos, with the exception of haploid morulae supplemented with IWR-1 (Table 4). With respect to cell allocation and lineage specification, embryos supplemented with CHIR99021 exhibited a similar number of SOX2-positive cells compared with IVF embryos and significantly higher levels than those observed in the IWR-1 and vehicle control (DMSO) groups (Table 5, Figure 3B), suggesting a beneficial effect of CHIR99021 on pluripotency in haploid parthenogenetic embryos.

Activin A (AA) has also been reported as an embryokine that enhances bovine embryo development to the blastocyst stage [28]. Therefore, the effects of CHIR99021 were directly compared with those of activin A using diploid parthenogenetic embryos as a control group. Activin A significantly increased the proportion of haploid parthenogenetic morulae progressing to the blastocyst stage, as well as the hatching rate, to levels comparable with diploid embryos and higher than those observed in the CHIR99021 and DMSO groups (Table 6).

**Figure 4:**
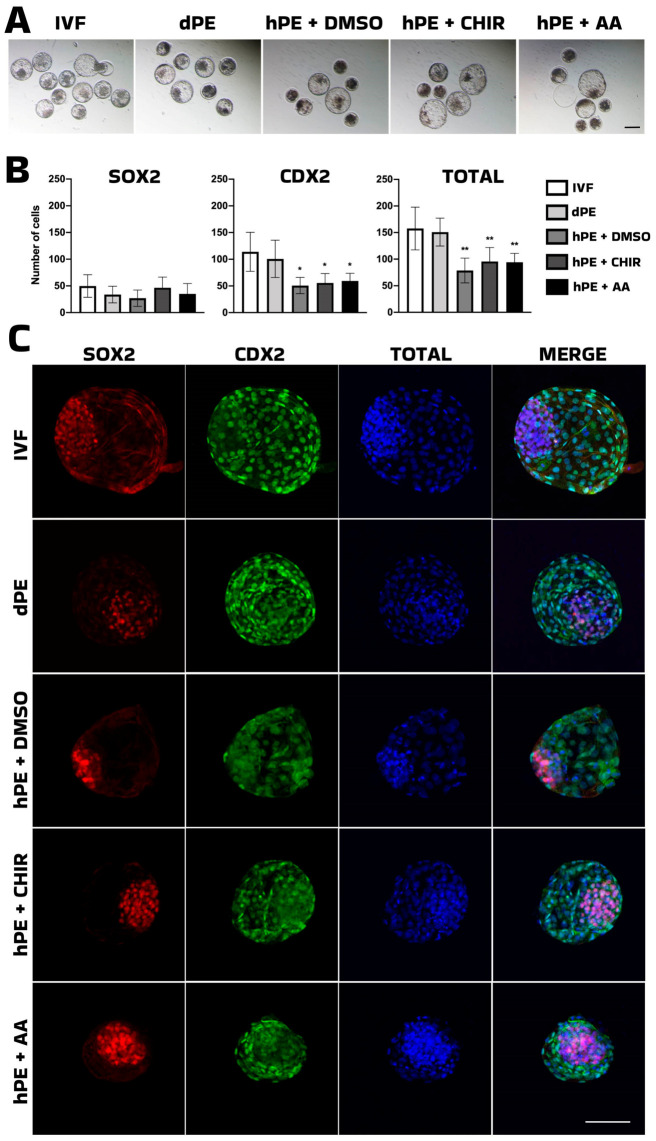
Effect of CHIR99021 and AA on the pluripotency markers in blastocysts. (**A**) Representative images of bovine in vitro-fertilized (IVF), diploid parthenogenetic embryos (dPE) and haploid parthenogenetic embryos (hPE) cultured from the morula stage onward in the presence of CHIR99021, activin A (AA), or vehicle control (DMSO). (**B**) Bar plot showing the quantification of pluripotency marker-positive cells analyzed by immunofluorescence in blastocysts, expressed according to cell lineage allocation and total cell number. (**C**) Representative fluorescent images of bovine in vitro-fertilized (IVF), diploid parthenogenetic embryos (dPE) and haploid parthenogenetic embryos (hPE) cultured from the morula stage onward in the presence of CHIR99021, activin A (AA), or vehicle control (DMSO). SOX2: red fluorescence channel depicting SRY-box transcription factor 2 (SOX2)-positive cells; CDX2: green fluorescence channel depicting Caudal-type homeobox 2 (CDX2)-positive cells; TOTAL: blue fluorescence channel indicating the total number of cells; Merge: composite images showing total cell number (blue), SOOX2-positive (red), and CDX2-positive cells (green). * Indicates statistically significant differences (* *p* < 0.05; ** *p* < 0.01). Scale bar = 100 μm.

Despite this positive effect on development, no differences were detected in the number of SOX2-positive cells among IVF, diploid parthenogenetic, and haploid parthenogenetic embryos, as assessed by immunofluorescence. In contrast, the number of CDX2-positive cells and the total cell number were higher in diploid parthenogenetic and IVF embryos (Table 7, Figure 4), suggesting that small molecule supplementation does not significantly enhance the proliferative capacity of the inner cell mass, while parthenogenetic haploidy imposes a persistent constraint on trophectoderm expansion.

### 3.3. Effects of CHIR99021 and Activin A on DNA Fragmentation

**Figure 5:**
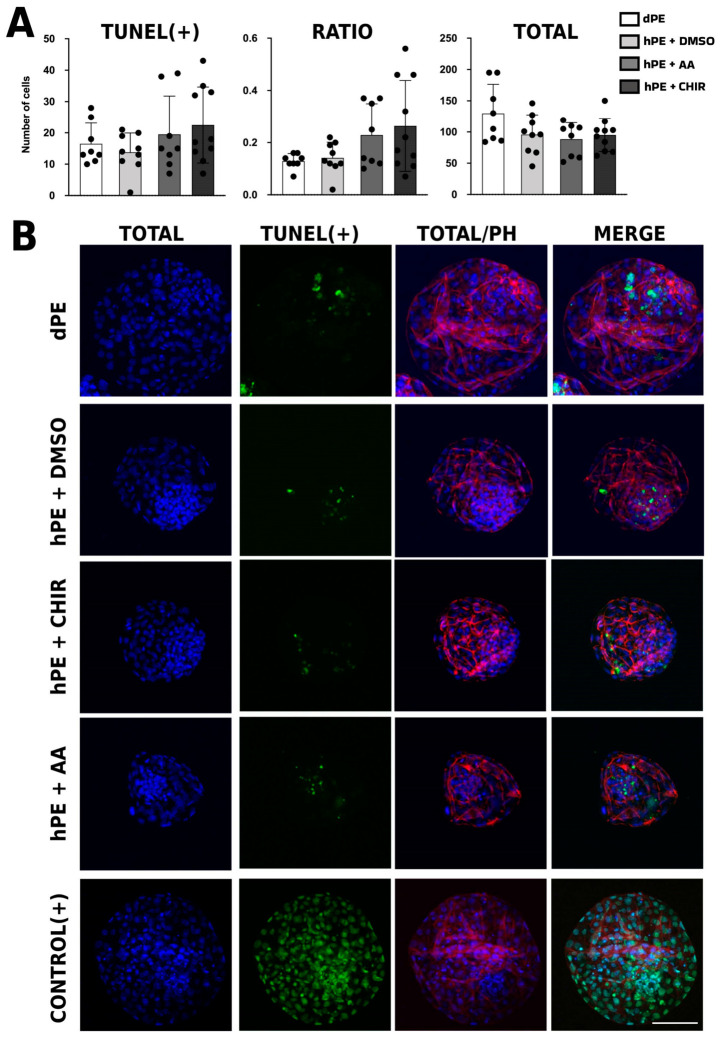
Quantification of TUNEL-positive cells in blastocysts. (**A**) Bar graph showing the quantification of TUNEL-positive cells in blastocysts, expressed as the absolute number of TUNEL-positive cells (TUNEL+), the proportion of TUNEL-positive cells relative to the total cell number (RATIO), and the total cell number per blastocyst (TOTAL). (**B**) Representative fluorescence images of TUNEL staining in bovine diploid parthenogenetic embryos (dPE) and haploid parthenogenetic embryos (hPE) cultured from the morula stage onward in the presence of CHIR99021, activin A (AA), or vehicle control (DMSO). TOTAL: Blue fluorescence channel indicating the total number of cells. TUNEL+: Green fluorescence channel indicating TUNEL-positive cells. TOTAL/PH: Merged images showing total cell number (blue) combined with cytoskeletal staining (red) using phalloidin 633. Merge: Composite images showing total cell number (blue), cytoskeletal staining (red; phalloidin 633), and TUNEL-positive cells (green). Scale bar = 100 μm.

Furthermore, morula-stage supplementation with CHIR99021 or activin A did not result in statistically significant differences in DNA fragmentation among groups (*p* = 0.07). Nevertheless, a clear tendency toward a higher proportion of cells with fragmented DNA was observed in the treated groups compared with diploid parthenogenetic embryos (dPE) and vehicle-treated haploid controls (hPE) (Figure 5), suggesting that pathway modulation may not fully mitigate apoptotic susceptibility in haploid embryos.

### 3.4. Effects of Activin A on Pluripotency-Associated Transcripts and Outgrowth Establishment Under Feeder-Free Conditions

**Figure 6:**
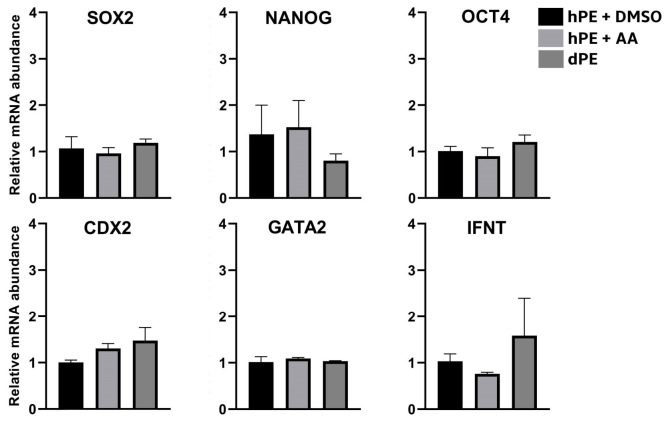
Relative mRNA abundance of SOX2, NANOG, OCT4, CDX2, GATA2, and IFNT in haploid parthenogenetic embryos cultured in the presence of Activin A (hPE + AA) or vehicle control (hPE + DMSO), compared with diploid parthenogenetic embryos (dPE). Gene expression levels were normalized to SF3A and β-actin genes and expressed relative to the control group. Ct values for all analyzed genes have been included as Supplementary Materials (Table S2). Data are presented as mean ± SEM.

Given that activin A (AA) supplementation enhanced progression to the blastocyst stage and achieved hatching rates comparable to diploid controls, its effects on mRNA levels of pluripotency-associated genes and post-blastocyst developmental potential were further investigated. No significant differences in transcripts levels were observed among groups (*p* > 0.05) (Figure 6). The Ct values for all analyzed transcripts have been included as Supplementary Materials (Table S2).

**Figure 7:**
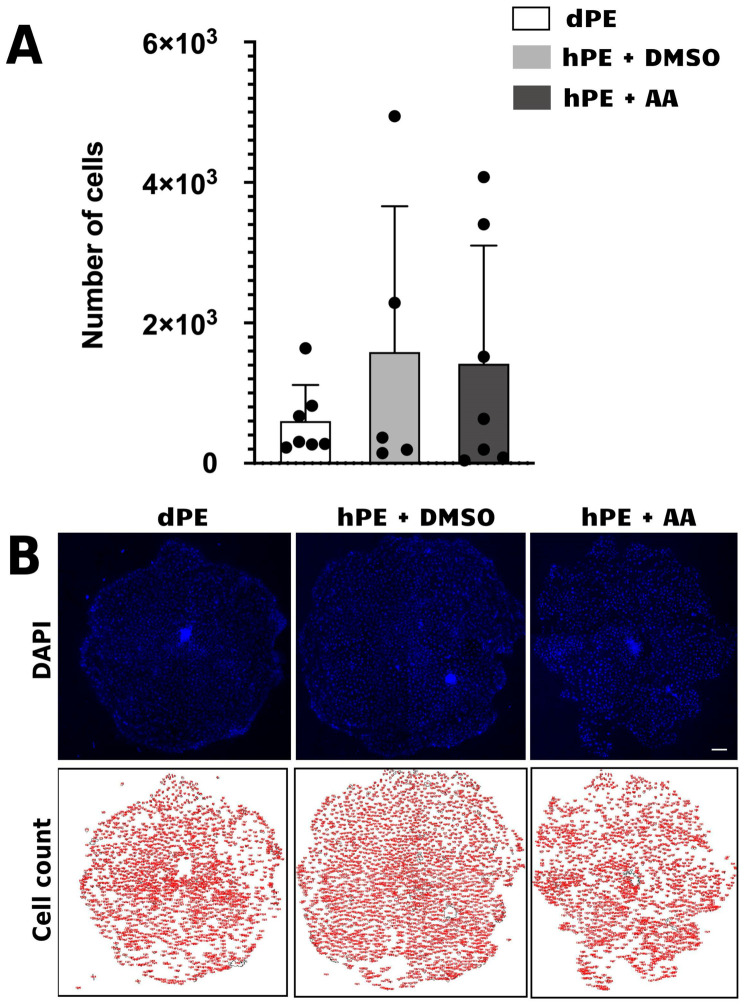
Quantification of cell number in embryonic outgrowths. (**A**) Bar graph showing the quantification of total cell number in embryonic outgrowths derived from diploid parthenogenetic embryos (dPE) and haploid parthenogenetic embryos (hPE) cultured from the morula stage (day 5) to the blastocyst stage (day 8) in the presence of activin A (AA) or vehicle control (DMSO). (**B**) Representative fluorescence (DAPI) images and corresponding outline visualization (cell count) used for quantification with ImageJ in bovine embryonic outgrowths derived from diploid parthenogenetic embryos (dPE) and haploid parthenogenetic embryos (hPE) cultured from the morula stage onward in the presence of activin A (AA) or vehicle control (DMSO). Scale bar = 100 μm.

Similarly, post-blastocyst developmental potential was assessed by evaluating embryonic outgrowth formation on vitronectin-coated wells in the presence of small molecules associated with stem cell derivation. This analysis revealed substantial variability in outgrowth development potential among replicates and detected no significant differences (*p* > 0.05) between AA-treated and untreated haploid parthenogenetic embryos, suggesting that AA supplementation does not confer additional advantages for post-blastocyst outgrowth formation under the conditions evaluated (Figure 7). Altogether, these results confirm that Activin A supplementation does not affect the expression levels of pluripotency-associated transcripts nor outgrowth development.

## 4. Discussion

The present study aimed to determine whether modulation of key signaling pathways could improve the developmental competence and lineage specification of bovine haploid parthenogenetic embryos (bhPE) under serum-free conditions. Overall, our findings indicate that although specific small molecules can enhance certain developmental parameters, none of the treatments tested were sufficient to overcome the intrinsic limitations imposed by haploid genome constitution.

As an initial step, we evaluated the replacement of fetal calf serum (FCS) with insulin–transferrin–selenium (ITS) to establish a chemically defined culture system. The use of fetal bovine serum (FBS) in embryo culture is known to introduce batch-to-batch variability due to its undefined and complex composition. In contrast, ITS is a well-characterized supplement that has been widely used as a serum substitute in cell and embryo culture systems. ITS supports cell proliferation, metabolism, and survival while reducing reliance on undefined serum components [29,30].

Pioneering studies have demonstrated that defined culture media supplemented with ITS can support in vitro development of bovine embryos produced by somatic cell nuclear transfer [31] and IVF [32]. Moreover, ITS supplementation has been successfully applied in various mammalian embryonic models [33,34,35], supporting its suitability as a serum-free alternative. In the present study, ITS supplementation supported normal developmental progression and pluripotency-associated marker expression in both IVF-derived and diploid parthenogenetic embryos. These findings indicate that ITS provides adequate trophic support in the absence of serum, without introducing detectable bias in developmental outcomes or lineage specification. Importantly, the establishment of this defined culture system enabled subsequent evaluation of signaling pathway modulators under controlled conditions, minimizing potential confounding effects associated with serum-derived factors.

The initial analysis of small molecules to identify factors capable to boost either competence or pluripotency of parthenogenetic haploid embryos showed that inhibition of SMAD-dependent TGF-β signaling using A83-01 from the morula stage onward did not improve blastocyst formation or morphology in bhPE. Although TGF-β pathway inhibition has been associated with enhanced pluripotency maintenance in stem cell systems [18,36], its modulation at post-compaction stages was insufficient to enhance lineage allocation in haploid embryos.

Next, modulation of the WNT pathway revealed a more nuanced response. CHIR99021, a GSK3β inhibitor and WNT agonist, increased the number of SOX2-positive cells in bhPE to levels comparable to IVF embryos, indicating a beneficial effect on pluripotency-associated lineage allocation in this model. Early studies found that the inhibition of GSK3 in conjunction with MEK from the zygote stage onward offered enhanced blastocyst development and expression of epiblast NANOG and SOX2 of bovine IVF embryos [37,38]. Similarly, inhibition of GSK3 and MAP2K improved blastocyst morphology and expression of pluripotency-associated genes (FGF4 and NANOG) [39]. In contrast, IWR-1, a WNT pathway antagonist, did not produce similar effects. Although the proportion of morulae progressing to the blastocyst stage was comparable to that of the IVF group, no improvements were observed in total cell number or lineage allocation. Tankyrase inhibitors, including IWR-1, have been reported as essential components for the derivation and maintenance of livestock embryonic stem cells (ESCs) [16]. However, in a subsequent study, the same group demonstrated that bovine ESCs exposed to IWR-1 exhibited reduced protein abundance of key HIPPO pathway components, including TEAD4 and YAP1, along with decreased expression of canonical YAP/TEAD target genes such as CYR61. These findings suggest that while IWR-1 may support ESC stabilization under specific culture conditions, its modulation of WNT and HIPPO signaling does not necessarily enhance lineage allocation in haploid embryos at the preimplantation stage.

We next evaluated the effects of Activin A supplementation in comparison with CHIR99021. The developmental progression of CHIR99021-treated haploid embryos remained inferior to diploid parthenogenetic control. Thus, while WNT activation may enhance aspects of inner cell mass specification, it does not restore global developmental competence. On the other hand, activin A (AA), a member of the TGF-β superfamily, has previously been described as an embryokine that enhances bovine embryo development to the blastocyst stage [28]. In the present study, AA supplementation significantly improved blastocyst formation and hatching rates in haploid parthenogenetic embryos, demonstrating that its beneficial effects extend to uniparental embryos. Interestingly, despite the clear improvement in developmental progression, AA supplementation did not alter the number of SOX2-positive cells in haploid embryos compared with control groups. This suggests that AA does not directly enhance SOX2-associated lineage specification within the inner cell mass (ICM), but rather promotes developmental competence through alternative mechanisms, potentially related to improved cell survival or developmental kinetics. In contrast, both the number of CDX2-positive cells and the total cell number remained higher in diploid parthenogenetic and IVF embryos compared with haploid embryos. These findings indicate that although AA improves blastocyst yield in haploid parthenotes, it does not restore proliferative capacity or lineage allocation to diploid levels, which agrees with a previous report indicating that AA improved blastocyst development of IVF embryos after supplementation at the morula stage [40]. The reduced total cell number observed in haploid parthenotes likely reflects intrinsic gene dosage limitations and epigenetic imbalances associated with uniparental genome constitution [10,11,12]. Taken together, these results suggest that Activin A enhances developmental efficiency without fundamentally correcting the intrinsic cellular and genomic constraints of haploid parthenogenetic embryos.

The analysis of DNA fragmentation revealed that the number of TUNEL-positive cells tended to be higher in both AA- and CHIR99021-treated haploid embryos compared with vehicle and diploid controls, suggesting that modulation of these signaling pathways does not mitigate the intrinsic susceptibility of haploid embryos to apoptotic stress. Rather, this trend may reflect persistent genomic instability and altered cell cycle regulation associated with haploidy, which cannot be fully compensated by activation of TGF-β or WNT signaling. Similar findings were reported by Madeja et al. [15], who observed increased apoptosis in 2i-treated blastocysts without concomitant changes in the transcript abundance of canonical apoptosis-related genes (BAX, BCL2, BAK, and the BAX/BCL2 ratio). Similarly, Trigal et al. [40] indicated that when AA was added for shorter periods activin increased apoptotic rates. These results suggest that elevated DNA fragmentation may occur independently of transcriptional regulation of classical apoptotic markers, potentially involving post-transcriptional mechanisms, mitochondrial dysfunction, replication stress, or alternative cell death pathways. Therefore, the possibility that non-canonical or transcriptionally uncoupled apoptotic pathways contribute to the increased DNA fragmentation observed in treated haploid embryos cannot be excluded.

Regarding the assessment of transcript levels associated with embryonic stemness (IFNT1, GATA2, CDX2, POU5F1, SOX2, and NANOG) [41], a trend toward increased CDX2 and IFNT expression was observed in dPE embryos, whereas NANOG levels tended to be slightly higher in hPE + AA embryos. CDX2 and IFNT are trophoblast-specific factors, and their upward trend was consistent with the immunofluorescence analysis. Similarly, although NANOG expression tended to be higher in haploid parthenotes treated with Activin A, none of these differences reached statistical significance. Functionally, NANOG is essential for the derivation and maintenance of the pluripotent epiblast and for the second lineage commitment [42,43], which is consistent with the presence of SOX2 in parthenogenetic embryos.

Although Activin A (AA) improved blastocyst formation and hatching rates in haploid parthenogenetic embryos, this benefit did not translate into enhanced outgrowth derivation, indicating that AA treatment during blastocyst formation does not confer sustained advantages beyond the blastocyst stage. The high variability and absence of improved outgrowth formation further support the notion that intrinsic limitations associated with haploid genome constitution, including embryonic quality, gene dosage imbalance and epigenetic dysregulation, remain uncorrected despite modulation of TGF-β signaling [9,13,44].

## 5. Conclusions

Collectively, these data reinforce the concept that improvements in blastocyst yield do not necessarily reflect restoration of intrinsic developmental competence. Our findings suggest that pathway modulation can influence lineage allocation dynamics but does not compensate for the broader genomic constraints imposed by haploidy. Importantly, we report for the first time the derivation of embryonic outgrowths from bovine parthenogenetic embryos in vitronectin-coated wells. Future research will be focused on the use of a combination of small molecules to modulate embryonic pluripotency as well as derivation of bovine uniparental ESCs under feeder-free conditions.

## Figures and Tables

**Table 1:** Effects of serum-free culture medium on developmental competence, cell number and expression of transcription factors associated with pluripotency of bovine embryos.

Group		Embryo Development						Embryo Morphology						Immunostaining (Mean ± S.D.)				
		Oocytes	n	Cleaved Embryos (No. and %)		Blastocyst (No. and %)		Early	%	Expanded	%	Hatched/Hatching	%	SOX2	CDX2	Total Cell	n	No. of Embryos
dPE	SFC	118	4	82	69%	44	37%	25	57%	18	41%	1	2%	34 ± 11	81 ± 17	120 ± 20	3	12
	FBS	150	5	114	76%	48	32%	29	60%	15	31%	4	8%	25 ± 13	93 ± 32	121 ± 36	3	10
IVF	SFC	166	5	130	78%	40	24%	28	70%	9	23%	3	8%	46 ± 10	97 ± 11	142 ± 18	3	10
	FBS	202	6	154	76%	44	22%	32	73%	6	14%	6	14%	43 ± 8	108 ± 16	151 ± 42	3	9

dPE: diploid parthenogenetic embryo; IVF: in vitro fertilization; SFC: serum-free culture; FBS: fetal bovine serum culture; SOX2: SRY-box transcription factor 2; CDX2: Caudal-type homeobox 2; (n): biological replicates.

**Table 2:** Effects of A83-01 supplementation of the culture medium from the morula stage onward on the developmental competence of haploid parthenogenetic embryos.

Group	Embryo Development									Blastocyst Morphology							
	Oocytes	(n)	Cleaved Embryos (No. and %)		Morulas (No. and %)		Blastocyst (No. and %)		Blasotcyst/Morulas (%)	Early	%	Expanded	%	Hatched	%	n	Blastos (No.)
hPE	435	6	373	86%	99	23%	71	16%	72%	54	76%	14	20%	3	4%	12	71
+DMSO					50	n.a.	35	n.a.	70%	27	77%	7	20%	1	3%	6	35
+A83					49	n.a.	36	n.a.	73%	27	75%	7	19%	2	6%	6	36

Embryonic development was assessed at the cleavage stage on day 2 (48 h), at the morula stage at day 5 (120 h) and at the blastocyst stage on day 8 (192 h) post-fertilization. hPE: haploid parthenogenetic embryo; DMSO: dimetilsulfoxide; A83: A83-01 ALK inhibitor; (n): biological replicates. Blastocyst/morula: proportion of morulae developing into blastocysts. n.a.: cleaving and morula’s rates were not included because DMSO and CHIR99021 treatment initiated at Day 5 of culture.

**Table 3:** Effects of A83-01 supplementation on the number of cells and pluripotency markers of parthenogenetic haploid blastocysts.

Group	SOX2	CDX2	Total	n	No. of Embryos
IVF	39 ± 9	86 ± 21	140 ± 24	4	11
hPE					
+DMSO	21 ± 19 *	66 ± 25	99 ± 42	4	9
+A83	16 ± 13 ***	62 ± 27	99 ± 45 *	4	12

IVF: in vitro-fertilized embryos; hPE: haploid parthenogenetic embryos; DMSO: dimetilsulfoxide; A83: A83-01 ALK inhibitor; (n): biological replicates. * Indicates statistically significant differences (* *p* < 0.05, *** *p* < 0.001).

**Table 4:** Effects of CHIR99021 or IWR1 supplementation of the culture medium from the morula stage onward on the developmental competence of haploid parthenogenetic embryos.

Group	Embryo Development									Blastocyst Morphology							
	Oocytes	(n)	Cleaved Embryos (No. and %)		Morulas (No. and %)		Blastocyst (No. and %)		Blasotcyst/Morulas (%)	Early	%	Expanded	%	Hatched	%	n	Blastos (No.)
hPEs	1091	9	904	83%	246	23%	167	15%	68% ^a^	136	81%	27	16% ^ab^	4	2%	9	167
+DMSO					85	n.a.	59	n.a.	69% ^a^	46	78%	11	19% ^ab^	0	0%	9	59
+CHIR					77	n.a.	48	n.a.	62% ^a^	37	77%	12	25% ^a^	1	2%	9	48
+IWR1					84	n.a.	60	n.a.	71% ^ab^	53	88%	4	7% ^b^	3	5%	9	60
IVF	296	8	244	82%	99	33% ***	80	27% ***	81% ^b^	60	75%	12	15% ^ab^	8	10%	8	80

Embryonic development was assessed at cleavage (day 2, 48 h), morula (day 5, 120 h), and blastocyst (day 8, 192 h) stages of embryo culture. hPE: haploid parthenogenetic embryo; DMSO: dimethyl sulfoxide; CHIR: CHIR99021 (GSK-3 inhibitor); IWR1: IWR-1-endo WNT pathway inhibitor; n: biological replicates. Blastocyst/morula indicates the proportion of morulae progressing to the blastocyst stage. Different superscripts within the same column indicate statistically significant differences (*** *p* < 0.001). n.a.: cleaving and morula’s rates were not included because DMSO and CHIR99021 treatment initiated at Day 5 of culture.

**Table 5:** Effects of CHIR99021 or IWR1 supplementation of the culture medium from the morula stage onward on number of cells and pluripotency markers of parthenogenetic haploid morulae.

Group	SOX2	CDX2	Total	n	No. of Embryos
IVF	37 ± 14 ^a^	106 ± 32	156 ± 42	6	22
hPE					
+DMSO	19 ± 16 ^b^	92 ± 39	127 ± 42	5	19
+CHIR	33 ± 16 ^ac^	93 ± 28	135 ± 34	5	18
+IWR1	18 ± 13 ^b^	105 ± 37	125 ± 41	5	13

IVF: in vitro-fertilized embryos; hPE: haploid parthenogenetic embryo; DMSO: dimethyl sulfoxide; CHIR: CHIR99021 (GSK-3 inhibitor); IWR1: IWR-1-endo WNT pathway inhibitor; n: biological replicates. Different superscripts within the same column indicate statistically significant differences (*p* < 0.05).

**Table 6:** Effects of CHIR99021 and Activin A supplementation of the culture medium from the morula stage onward on the developmental competence of haploid parthenogenetic embryos.

Group	Embryo Development									Blastocyst Morphology							
	Oocytes	(n)	Cleaved Embryos (No. and %)		Morulas (No. and %)		Blastocyst (No. and %)		Blasotcyst/Morulas (%)	Early	%	Expanded	%	Hatched	%	n	Blastos (No.)
hPEs	633	6	490	77%	118	19%	76	12%	64% ^b^	50	66%	21	28%	5	7%	6	76
+DMSO					38	n.a.	22	n.a.	58% ^b^	16	73%	6	27%	0	0%	6	22
+CHIR					39	n.a.	23	n.a.	59% ^b^	14	61%	8	35%	1	4%	6	23
+AA					41	n.a.	31	n.a.	76% ^ab^	20	65%	7	23%	4	13%	6	31
dPE	136	4	115	85%	53	39% ***	44	32% ***	83% ^ab^	14	32% *	23	52% *	7	16%	4	44

Embryonic development was assessed at cleavage (day 2, 48 h), morula (day 5, 120 h), and blastocyst (day 8, 192 h) stages post-fertilization. hPE: haploid parthenogenetic embryo; DMSO: dimethyl sulfoxide; CHIR: CHIR99021 (GSK-3 inhibitor); AA: activin A; n: biological replicates. Blastocyst/morula indicates the proportion of morulae progressing to the blastocyst stage. (* *p* < 0.05; *** *p* < 0.001). Different superscripts within the same column indicate statistically significant differences (*p* < 0.05). n.a.: cleaving and morula’s rates were not included because DMSO and CHIR99021 treatment initiated at Day 5 of culture.

**Table 7:** Effects of CHIR99021 or Activin A supplementation of the culture medium from the morula stage onward on the number of cells and pluripotency markers of parthenogenetic haploid morulas.

Group	SOX2	CDX2	Total	n	No. of Embryos
IVF	50 ± 21	114 ± 37 **	158 ± 40 *	3	6
dPE	34 ± 16	101 ± 35 *	151 ± 26 *	4	11
hPE					
+DMSO	27 ± 15	51 ± 15	79 ± 23	4	10
+CHIR	46 ± 20	56 ± 17	96 ± 26	4	9
+AA	35 ± 20	59 ± 14	94 ± 17	3	7

IVF: in vitro-fertilized embryos; hPE: haploid parthenogenetic embryo; DMSO: dimethyl sulfoxide; CHIR: CHIR99021 (GSK-3 inhibitor); AA: Activin A; n: biological replicates. (* *p* < 0.05; ** *p* < 0.01).

## Data Availability

The data supporting the findings of this study are available from the corresponding author upon reasonable request.

## Supplementary Materials

The following supporting information can be downloaded at https://www.mdpi.com/article/10.3390/ani16101517/s1. Table S1: Details of primers used for RT-qPCR analysis; Table S2: Ct values for all analyzed transcripts.

## Abbreviations

The following abbreviations are used in this manuscript:

DMSO	Dimethyl sulfoxide
TUNEL	Terminal deoxynucleotidyl transferase dUTP nick-end labeling
IVM	in vitro maturation
COCs	Cumulus–oocyte complexes
CO_2_	Carbon dioxide

## References

[1] Cruz N.T.D., Wilson K.J., Cooney M.A., Tecirlioglu R.T., Lagutina I., Galli C., Holland M.K., French A.J. (2008). Putative imprinted gene expression in uniparental bovine embryo models. Reprod. Fertil. Dev..

[2] Hu M., Zhao Z., TuanMu L.-C., Wei H., Gao F., Li L., Ying J., Zhang S. (2015). Analysis of imprinted gene expression and implantation in haploid androgenetic mouse embryos. Andrologia.

[3] Leng L., Sun J., Huang J., Gong F., Yang L., Zhang S., Yuan X., Fang F., Xu X., Luo Y. (2019). Single-Cell Transcriptome Analysis of Uniparental Embryos Reveals Parent-of-Origin Effects on Human Preimplantation Development. Cell Stem Cell.

[4] Kokubu C., Takeda J. (2014). When Half Is Better Than the Whole: Advances in Haploid Embryonic Stem Cell Technology. Cell Stem Cell.

[5] Bai M., Han Y., Wu Y., Liao J., Li L., Wang L., Li Q., Xing W., Chen L., Zou W. (2019). Targeted genetic screening in mice through haploid embryonic stem cells identifies critical genes in bone development. PLoS Biol..

[6] Leeb M., Wutz A. (2011). Derivation of haploid embryonic stem cells from mouse embryos. Nature.

[7] Zhong C., Zhang M., Yin Q., Zhao H., Wang Y., Huang S., Tao W., Wu K., Chen Z.-J., Li J. (2016). Generation of human haploid embryonic stem cells from parthenogenetic embryos obtained by microsurgical removal of male pronucleus. Cell Res..

[8] Yang H., Liu Z., Ma Y., Zhong C., Yin Q., Zhou C., Shi L., Cai Y., Zhao H., Wang H. (2013). Generation of haploid embryonic stem cells from Macaca fascicularis monkey parthenotes. Cell Res..

[9] Latham K.E., Akutsu H., Patel B., Yanagimachi R. (2002). Comparison of Gene Expression During Preimplantation Development Between Diploid and Haploid Mouse Embryos. Biol. Reprod..

[10] Latham K.E., Doherty A.S., Scott C.D., Schultz R.M. (1994). Igf2r and Igf2 gene expression in androgenetic, gynogenetic, and parthenogenetic preimplantation mouse embryos: Absence of regulation by genomic imprinting. Genes Dev..

[11] Ogawa H., Wu Q., Komiyama J., Obata Y., Kono T. (2006). Disruption of parental-specific expression of imprinted genes in uniparental fetuses. FEBS Lett..

[12] Hu M., TuanMu L.-C., Wei H., Gao F., Li L., Zhang S. (2015). Development and imprinted gene expression in uniparental preimplantation mouse embryos in vitro. Mol. Biol. Rep..

[13] Aguila L., Suzuki J., Hill A.B.T., García M., de Mattos K., Therrien J., Smith L.C. (2021). Dysregulated Gene Expression of Imprinted and X-Linked Genes: A Link to Poor Development of Bovine Haploid Androgenetic Embryos. Front. Cell Dev. Biol..

[14] Xiao Y., Sosa F., Ross P.J., Diffenderfer K.E., Hansen P.J. (2021). Regulation of NANOG and SOX2 expression by activin A and a canonical WNT agonist in bovine embryonic stem cells and blastocysts. Biol. Open.

[15] Madeja Z.E., Warzych E., Pawlak P., Lechniak D. (2019). Inhibitor mediated WNT and MEK/ERK signalling affects apoptosis and the expression of quality related genes in bovine in vitro obtained blastocysts. Biochem. Biophys. Res. Commun..

[16] Xiao Y., Amaral T.F., Ross P.J., Soto D.A., Diffenderfer K.E., Pankonin A.R., Jeensuk S., Tríbulo P., Hansen P.J. (2021). Importance of WNT-dependent signaling for derivation and maintenance of primed pluripotent bovine embryonic stem cells. Biol. Reprod..

[17] Warzych E., Pawlak P., Lechniak D., Madeja Z.E. (2020). WNT signalling supported by MEK/ERK inhibition is essential to maintain pluripotency in bovine preimplantation embryo. Dev. Biol..

[18] Pillai V.V., Koganti P.P., Kei T.G., Gurung S., Butler W.R., Selvaraj V. (2021). Efficient induction and sustenance of pluripotent stem cells from bovine somatic cells. Biol. Open.

[19] Valencia C., Pérez-García F., Aguila L., Felmer R., Arias M.E. (2023). Combined Exogenous Activation of Bovine Oocytes: Effects on Maturation-Promoting Factor, Mitogen-Activated Protein Kinases, and Embryonic Competence. Int. J. Mol. Sci..

[20] Park J., Oh H., Hong S., Jang G., Kim M., Lee B. (2008). Effects of Activin A on the In Vitro Development and mRNA Expression of Bovine Embryos Cultured in Chemically-Defined Two-Step Culture Medium. Reprod. Domest. Anim..

[21] Tribulo P., Leão B.C.d.S., Lehloenya K.C., Mingoti G.Z., Hansen P.J. (2017). Consequences of endogenous and exogenous WNT signaling for development of the preimplantation bovine embryo. Biol. Reprod..

[22] Tríbulo P., Jumatayeva G., Lehloenya K., Moss J.I., Negrón-Pérez V.M., Hansen P.J. (2018). Effects of sex on response of the bovine preimplantation embryo to insulin-like growth factor 1, activin A, and WNT7A. BMC Dev. Biol..

[23] Bogliotti Y.S., Wu J., Vilarino M., Okamura D., Soto D.A., Zhong C., Sakurai M., Sampaio R.V., Suzuki K., Izpisua Belmonte J.C. (2018). Efficient derivation of stable primed pluripotent embryonic stem cells from bovine blastocysts. Proc. Natl. Acad. Sci. USA.

[24] Aguila L., Nociti R.P., Sampaio R.V., Therrien J., Meirelles F.V., Felmer R.N., Smith L.C. (2023). Haploid androgenetic development of bovine embryos reveals imbalanced WNT signaling and impaired cell fate differentiation. Biol. Reprod..

[25] Su Y., Zhao R., Fang Y., Renxiu M., Li G., Jin L., Liu J., Yang Z., Li N., Zhu J. (2026). Bovine formative embryonic stem cell plasticity in embryonic and extraembryonic differentiation. Stem Cells.

[26] Sampaio R.V., Sangalli J.R., De Bem T.H.C., Ambrizi D.R., del Collado M., Bridi A., de Ávila A.C.F.C.M., Macabelli C.H., de Jesus Oliveira L., da Silveira J.C. (2020). Catalytic inhibition of H3K9me2 writers disturbs epigenetic marks during bovine nuclear reprogramming. Sci. Rep..

[27] Pfaffl M.W., Tichopad A., Prgomet C., Neuvians T.P. (2004). Determination of stable housekeeping genes, differentially regulated target genes and sample integrity: BestKeeper—Excel-based tool using pair-wise correlations. Biotechnol. Lett..

[28] Kannampuzha-Francis J., Tribulo P., Hansen P.J. (2017). Actions of activin A, connective tissue growth factor, hepatocyte growth factor and teratocarcinoma-derived growth factor 1 on the development of the bovine preimplantation embryo. Reprod. Fertil. Dev..

[29] Chua K., Aminuddin B., Fuzina N., Ruszymah B. (2005). Insulin-Transferrin-Selenium Prevent Human Chondrocyte Dedifferentiation and Promote the Formation of High Quality Tissue Engineered Human Hyaline Cartilage. Eur. Cells Mater..

[30] Liu X., Zhang T., Wang R., Shi P., Pan B., Pang X. (2019). Insulin-Transferrin-Selenium as a Novel Serum-free Media Supplement for the Culture of Human Amnion Mesenchymal Stem Cells. Ann. Clin. Lab. Sci..

[31] Wang L.-J., Xiong X.-R., Zhang H., Li Y.-Y., Li Q., Wang Y.-S., Xu W.-B., Hua S., Zhang Y. (2012). Defined media optimization for in vitro culture of bovine somatic cell nuclear transfer (SCNT) embryos. Theriogenology.

[32] Wydooghe E., Heras S., Dewulf J., Piepers S., Van den Abbeel E., De Sutter P., Vandaele L., Van Soom A. (2014). Replacing serum in culture medium with albumin and insulin, transferrin and selenium is the key to successful bovine embryo development in individual culture. Reprod. Fertil. Dev..

[33] Le B.A.M., Nguyen L.B.L., Lam C.T., Nguyen N.T., Nguyen N.T.V., Nguyen V.T., Bui H.T. (2025). Improving porcine in vitro blastocyst development using fetal bovine serum, amino acids, and insulin-transferrin-selenium. J. Reprod. Dev..

[34] Duque Rodriguez M., Cittadini C.O., Teplitz G.M., De Stefano A., Lombardo D.M., Salamone D.F. (2021). Canine IVM With SOF Medium, Insulin-Transferrin-Selenium, and Low O_2_ Tension Improves Oocyte Meiotic Competence and Decreases Reactive Oxygen Species Levels. Front. Cell Dev. Biol..

[35] Karami N., Taei A., Hassani S.N., Alizadeh N., Eftekhari-Yazdi P., Hassani F. (2025). The effects of insulin–transferrin–selenium (ITS) and CHIR99021 on the development of pre-implantation human arrested embryos in vitro. Sci. Rep..

[36] Kim D., Roh S. (2021). Strategy to Establish Embryo-Derived Pluripotent Stem Cells in Cattle. Int. J. Mol. Sci..

[37] Madeja Z.E., Sosnowski J., Hryniewicz K., Warzych E., Pawlak P., Rozwadowska N., Plusa B., Lechniak D. (2013). Changes in sub-cellular localisation of trophoblast and inner cell mass specific transcription factors during bovine preimplantation development. BMC Dev. Biol..

[38] Madeja Z.E., Hryniewicz K., Orsztynowicz M., Pawlak P., Perkowska A. (2015). WNT/β-Catenin Signaling Affects Cell Lineage and Pluripotency-Specific Gene Expression in Bovine Blastocysts: Prospects for Bovine Embryonic Stem Cell Derivation. Stem Cells Dev..

[39] McLean Z., Meng F., Henderson H., Turner P., Oback B. (2014). Increased MAP Kinase Inhibition Enhances Epiblast-Specific Gene Expression in Bovine Blastocysts. Biol. Reprod..

[40] Trigal B., Gómez E., Díez C., Caamaño J.N., Martín D., Carrocera S., Muñoz M. (2011). In vitro development of bovine embryos cultured with activin A. Theriogenology.

[41] Soto Pablo J., Ross D.A. (2016). Pluripotent stem cells and livestock genetic engineering. Transgenic Res..

[42] Springer C., Zakhartchenko V., Wolf E., Simmet K. (2021). Hypoblast Formation in Bovine Embryos Does Not Depend on NANOG. Cells.

[43] Ortega M.S., Kelleher A.M., O’neil E., Benne J., Cecil R., Spencer T.E. (2020). NANOG is required to form the epiblast and maintain pluripotency in the bovine embryo. Mol. Reprod. Dev..

[44] Wu Q., Kumagai T., Kawahara M., Ogawa H., Hiura H., Obata Y., Takano R., Kono T. (2006). Regulated expression of two sets of paternally imprinted genes is necessary for mouse parthenogenetic development to term. Reproduction.

